# Comparative study of crystallization kinetics and phase segregation of triple cation and methyl­ammonium lead iodide perovskites on moisture probing using synchrotron X-ray based radiation

**DOI:** 10.1107/S1600577524010695

**Published:** 2025-01-01

**Authors:** Miller Shatsala, Stellah Wanyonyi, Celline Awino, Maxwell Mageto, Hussein Golicha

**Affiliations:** a Department of Physics Masinde Muliro University of Science and Technology Kakamega Kenya; b Department of Pure and Applied Chemistry Masinde Muliro University of Science and Technology Kakamega Kenya; NSRRC, Taiwan

**Keywords:** 3D perovskite, crystallization, phase segregation, GWAXS, micro-diffraction

## Abstract

3D mixed perovskites have achieved substantial success in boosting solar cell efficiency, but the complicated perovskite crystal formation pathway remains mysterious. Here we present detailed crystallization kinetics of mixed perovskites FA_0.83_MA_0.17_Pb(I_0.83_Br_0.17_)_3_, where FA is formamidinium and MA is methylammonium, with the addition of Cs^+^ to form a triple cation perovskite (3-CAT), in a comparison with the perovskite building block MAPbI_3_ (MAPI) via static grazing-incidence wide-angle X-ray scattering (GIWAXS) and micro-diffraction measurements. Spin-coated films produced α-perovskite peaks with no PbI_2_ or δ-intermediate phases, which was a promising result for the 3-CAT perovskite from micro-diffraction measurements. However, the 3-CAT did not remain stable on probing with varied relative humidity (RH) conditions as segregation back to the δ-intermediate and PbI_2_ phase after 10 s of exposure to an RH value of 11% was found to occur from the GIWAXS results. When RH levels were elevated to over 100%, segregation peaks of PbI_2_ and δ-intermediate (2H, 4H and 6H) became conspicuous as the α-phase intensity diminished, unlike for MAPI that remains relatively stable. The possible cause of this is hydro­philic bonds that form between the 3-CAT crystals and the small annealing window of the best composition perovskite (5% Cs^+^) film.

## Abstract 2

Synchrotron techniques have been used in the analysis of crystal structure and compositions of materials, in particular for analyzing the crystallization kinetics and phase segregation of perovskite solar films on humidity probing.

## 1. Introduction

Solar cells are classified into first (monocrystalline and polycrystalline), second (amorphous silicon, CdTe and CIGS cells) and third (thin film and developing technologies including perovskites) generations (Goswami, 2021 ▸). The ‘hybrid’ tandem approach, where perovskites provide a boost in efficiency, presents an exciting path to delivering higher-efficiency panels without significant additional costs (Snaith, 2013 ▸; Brenner *et al.*, 2016 ▸). This may be the most direct pathway to commercializing perovskite-based photovoltaics, and also one of the fastest routes to realizing a step improvement in commercially available c-Si module efficiencies. For this reason, the perovskite–Si tandem has been the focus of research and recently reached efficiencies of 23.6% and 26.4% in two-terminal and four-terminal configurations, respectively, approaching a world record c-Si performance of 26.6% (Yu *et al.*, 2018 ▸). However, to achieve this noble idea, a good performing perovskite (in terms of efficiency and stability) has to be determined or else the whole process will be a far-fetched idea.

Recently, much effort has been channeled towards developing and controlling deposition methods in order to make solar modules available at low cost, with easy fabrication, and high efficiency (Li *et al.*, 2018 ▸; Chen *et al.*, 2015 ▸). However, thermal annealing, the use of additives, modifications using concentration, changing solvents and thermal casting (Tavakoli *et al.*, 2015 ▸; Min *et al.*, 2022 ▸; Tian *et al.*, 2019 ▸; Cao *et al.*, 2019 ▸) have all been reported to control perovskite structure, grain size, surface coverage, crystallinity and stability. To further understand the effects of these treatments, it is important to consider their effects on nucleation and growth which in turn influence the size, purity, morphology and crystal structure. These will make it possible to develop reproducible perovskite solar cells with optimized physical and morphological properties.

The one-step deposition method used in other investigations has faced difficulties in producing homogeneous morphologies (Cao *et al.*, 2019 ▸). Excess salt plays a crucial role in the crystallization by slowing down the process during annealing. However, the anti-solvent method – which is now the most commonly used – has shown remarkable improvements in the preparation of perovskite solar cells (PCSs) (Lee *et al.*, 2012 ▸). Chloro­benzene has been used before to remove excess dimethylformamide (DMF) in a perovskite solution (MAPbI_3_ in DMF, where MA is methylammonium) during the spin-coating process, rendering a homogeneous morphology and better performance in comparison with conventional deposition (Xiao *et al.*, 2014 ▸). In similar procedures, Jeon *et al.* (2014 ▸) mixed dimethyl sulfoxide (DMSO) and γ-butyrolactone (GBL) solvents and used toluene as an anti-solvent to achieve extremely dense and uniform films in which they found an intermediate phase of MAI–PbI_2_–DMSO (where MAI is methylammonium iodide). This phase was confirmed from MAPbI_3_ using DMF/DMSO as solvents by *in situ* grazing-incidence wide-angle X-ray scattering (GIWAXS) (da Silva *et al.*, 2020 ▸).

Pereyra *et al.* (2021 ▸) explain additive engineering and the role of the functional groups of these additives for defect passivation. The effect of the stability of PSCs under environmental conditions such as humidity, atmosphere, light irradiation (UV, visible) and heat has been presented by Liu *et al.* (2022 ▸) and the effect of moisture-triggered crystallization on caesium, MA and formamidinium (FA) triple-cation perovskite (Cs_0.05_FA_0.75_MA_0.20_)Pb(I_0.96_Br_0.04_)_3_ has also been discussed. A controllable moisture treatment for the intermediate perovskite may enhance mass transportation of organic salts, and help them enter the buried bottom of the films (Mahmood & Wang, 2020 ▸). It has further been mentioned that the process accelerates the quasi-solid–solid reaction between organic salts and PbI_2_, enables a spatially homogeneous intermediate phase, and translates to high-quality perovskites with much-suppressed defects.

Organic solar cells (OSCs) are reported to have been analyzed using GIWAXS and grazing-incidence small-angle X-ray scattering (GISAXS), concentrating on different pre- and post-processing conditions (solvent effect, solvent additive, solvent and thermal annealing), and the impact of the donor:acceptor ratio and molecular weight of semiconductor on microstructure (Mahmood & Wang, 2020 ▸). Therefore, synchrotron-radiation-based X-ray techniques allow structural characterization providing valuable information about the inner film morphology, *e.g.* wide-angle X-ray scattering (WAXS) probes length scales in the atomic range and thus yields crystallographic information about the sample (Schlipf & Müller-Buschbaum, 2017 ▸), while GIWAXS is a technique that is surface-sensitive (due to the grazing incidence used) and probes longer length scales, thus providing a full mesoscale approach to the problem of crystallization and the morphology of the samples (Erdemir *et al.*, 2009 ▸). Relevant information can be extracted from GIWAXS curves such as sizes, shapes, distances and correlations of particles (Li, Senesi & Lee, 2016 ▸). Such parameters are fundamental (Renaud *et al.*, 2009 ▸) in understanding the process of nucleation and growth.

Micro X-ray diffraction (micro-XRD) techniques are used to identify and quantify the various phases present in perovskite thin films (Tan & McNeill, 2022 ▸), analyze the texture of the films, and determine the micro-stress and macro-strain of the films. In addition, *in situ* and *operando* characterization of 3D mixed perovskites has achieved substantial success in boosting solar cell efficiency, but the complicated perovskite crystal formation pathway remains mysterious.

Detailed crystallization kinetics of mixed perovskites FA_0.83_MA_0.17_Pb(I_0.83_Br_0.17_)_3_ with addition of Cs^+^ to form a triple cation perovskite (3-CAT) is presented in a comparison with the perovskite building block MAPbI_3_ (MAPI) by static GIWAXS and micro-diffraction measurements. The films produced α-perovskite peaks with no PbI_2_ or δ-intermediate phases, which was a promising result for the 3-CAT perovskite from the micro-diffraction measurements. However, the 3-CAT did not remain stable on probing with varied relative humid (RH) conditions as segregation back to the δ-intermediate and PbI_2_ phase after 10 s of exposure to an RH value of 11% was found to occur from the GIWAXS results. When RH levels were elevated to over 100%, segregation peaks of PbI_2_ and δ-intermediate (2H, 4H and 6H) became conspicuous as the α-phase intensity diminished, unlike for MAPI that remains relatively stable. The possible cause of this is attributed to two factors: hydro­philic bonds which form between the 3-CAT crystals, and the small annealing window of the best composition perovskite (5% Cs^+^) film of perovskite.

This research seeks to address the influence of humidity on the crystallization kinetics and phase segregation of typical triple cations of Cs_0.05_(MA_0.17_FA_0.83_)_0.95_Pb (I_0.83_ Br_0.17_)_3_ and MAPbI_3_ on moisture probing of perovskite absorber film of solar cells using GIWAXS and micro-diffraction synchrotron techniques through variation of anti-solvent treatments. This is to help alleviate phase instability issues inherent in perovskite solar cells, which is a pathway to their commercialization.

## 2. Experimental

### 2.1. Synchrotron-based GIWAXS experiment

GIWAXS measurements were performed at the 7.3.3 beamline at the Advanced Light Source (ALS) of Lawrence Berkeley National Laboratory (LBNL), USA. The energy of the X-rays used was fixed at 10 keV with a current of 500 mA. The scattering signal was collected using a Dectris Pilatus3 2M detector. The beam wavelength was 0.124 nm, the energy bandwidth Δ*E*/*E* = 1% and beam size ≃ 300 µm (H) × 700 µm (W) (thin film size of 1.5 cm × 0.5 cm).

GIWAXS analysis was carried out at a sample-to-detector distance of 30 cm with an exposure time of 10 s, at an angle of 0.25° in order to obtain sufficient information of the diffraction patterns at the bulk of the perovskite sample, hence probing the crystal surface orientation of the sample. The 2D GIWAXS detector images were calibrated with silver behenate (AgB) and converted into *Q*-space and typical line cuts to form a graph of 1D intensity versus *Q* which allowed for more qualitative analysis of peak evolution during crystallization (Coffey *et al.*, 2023*a* ▸; Coffey *et al.*, 2023*b* ▸). *IgorPro* software was used for the data analysis.

### 2.2. Synchrotron-based X-ray micro-diffraction experiment

The synchrotron-based X-ray micro-diffraction experiment was conducted at beamline 12.3.2 at the ALS. The beamline has a superbend source, with an energy range of 6–22 keV and a wavelength range of 0.56–2.1 Å, with a high revolving power of 6000 radians. The beam size was approximately 1 µm at the sample position, which was set by a pair of Kirkpatrick–Baez (KB) mirrors that are elliptically bent and placed in a vacuum box. A 10 keV monochromatic X-ray beam was used to measure the structural behavior of the perovskite material. The monochromator and KB mirrors were placed inside a compact Plexiglas box filled with helium to improve thermal stability and reduce X-ray air scattering and absorption. The diffraction patterns were collected with a Pilatus3 1M detector. The sample is usually mounted in a 45° active geometry. The detector was placed on a vertical slide at approximately 35 mm from the sample area illuminated by the beam. This allows for the collection of a large solid angle of reciprocal space without having to move the detector.

### 2.3. Synchrotron-coupled RH

A humidifier was used to moisturize the chamber mounted on the GIWAXS stage. The custom-made partially transparent chamber has auxiliary provisions to which the humidity probe and the communication sensor are connected. The humidifier is fitted with a reader interface monitor that can make both humidity and temperature measurements. It has a water section connected to a hose tube that has a probe to the chamber. In twin with the hose tube, there is a communication sensor cable also connected to the helium chamber on the GIWAXS stage that constantly monitors the humidity environment in the chamber (on the film).

The system is controlled by a *LabVIEW* interface, whereby the relative humidity levels were reduced by purging the moisture in the chamber using helium; helium is used since we limited the oxygen content in the chamber which could have had an overall effect on the films. Measurements were taken at room temperature, 25°C ± 0.5°C. The RH values on the helium chamber were ramped from 0% to 110%. It took on average 20 min for the non-equilibrium conditions to be realized. It should be noted that, beyond the 100% humidity mark, non-equilibrium conditions set in and the air became supersaturated; with the absence of condensation nuclei in the chamber, the environment remained fairly vaporized.

### 2.4. Perovskite film fabrication

Perovskites such as Cs_0.05_(MA_0.17_FA_0.83_)_0.95_Pb(I_0.83_ Br_0.17_)_3_ and MAPbI_3_ were prepared by the reaction of inorganic precursor salts – CsI, PbBr_2_ and PbI_2_ – with an organic halide precursor solution – FAI and MA (where FAI is formamidinium iodide).

Inorganic precursor salts were prepared separately in different vials as follows: 50 mg of CsI was dissolved in 127.7 µl of DMSO; and 220 mg of PbBr_2_ was dissolved in 398.3 µl of DMF:DMSO 4:1 *v*/*v* ratio. Finally, 860 mg of PbI_2_ was dissolved in 1107.5 µl of DMF:DMSO 4:1 *v*/*v* ratio. The precursor solutions were heated at 150°C for 30 min in a nitro­gen-filled glovebox until fully dissolved. After that, 84.1 µl of CsI solution and 328.2 µl of PbBr_2_ solution were added to a vial containing PbI_2_ solution. The mixture was then stirred for 30 min in a nitro­gen-filled glovebox to form a ‘stock solution’ which was to be used in the next step of perovskite preparation.

Similarly, 45 mg of MAI and 217.9 mg of FAI were weighed in separate vials. 216.4 µl of stock solution was added to the vial containing MAI and 968.6 µl of the same stock solution to the vial containing FAI. The solutions in the two vials were allowed to dissolve for 90 min at room temperature in a nitro­gen-filled glovebox. To form 3-CAT, 144.7 µl of the solution in the vial of MAI was added to the solution in the vial containing FAI and again stirred for 90 min at room temperature inside the nitro­gen-filled glovebox. 19.4 µl of PbI_2_ excess was added to the vial containing the solution of FAI and stirred again for 90 min. Finally, 41.41 mg of MACl was carefully added to the vial containing the solution of FAI and then again stirred for 90 min. To prepare Cs_0.05(_FA_0.83_MA_0.17_)_0.95_Pb(I_0.83_Br_0.17_)_3_ films, 50 µl of the precursor solution was spin coated onto a 15 mm × 15 mm substrate at 3000 r.p.m. for 30 s. 210 µl of ethyl-acetate or 210 µl of chloro­benzene were used as anti-solvent and introduced after 25 s prior to the end of spin coating to evaporate the solvent from the film. The films were annealed at 100°C for 15 min on a hot plate at 1000 r.p.m.

1 *M* MAPI was prepared in a stoichiometric ratio of 1:1 by dissolving 1 *M* MAI and 1 *M* PbI_2_ in a solvent of DMF:DMSO 6:1 *v*:*v*. The precursor solution was stirred at 60°C in a nitro­gen-filled glovebox overnight. 50 µl of the precursor solution was spin coated onto a 15 mm × 15 mm substrate at 4000 r.p.m. for 25 s (4000 r.p.m. s^−1^ acceleration). 210 µl ethyl acetate or chloro­benzene was dripped into the spinning substrate after 20 s and annealed directly for 10 min at 100°C.

## 3. Results and discussion

### 3.1. Effects of varying the concentration of caesium in 3-CAT

**Figure 1:**
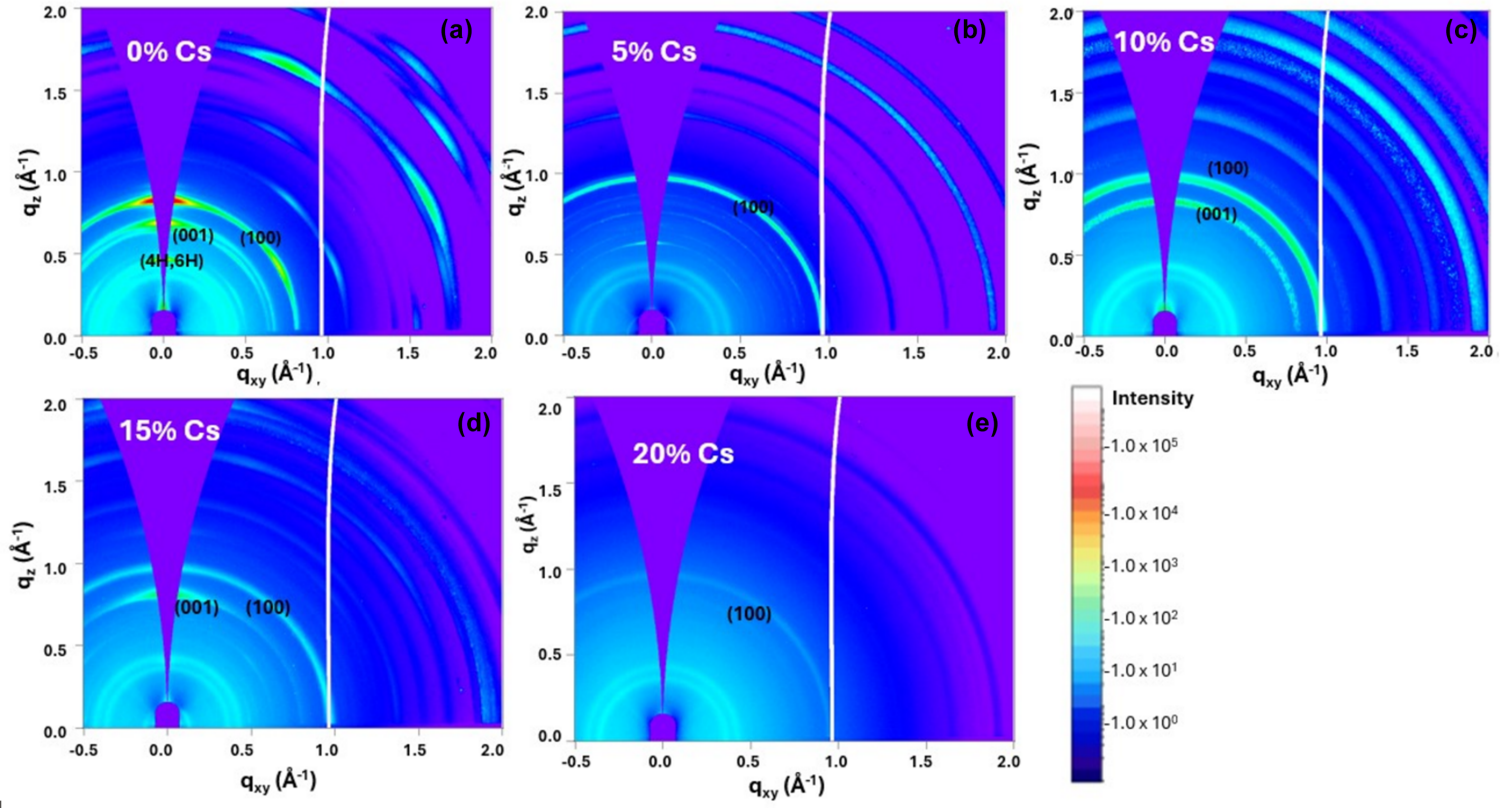
GIWAXS maps of 3-CAT with varying caesium concentration: (*a*) 0% Cs, (*b*) 5% Cs, (*c*) 10% Cs, (*d*) 15% Cs and (*e*) 20% Cs.

Initially we fabricated 3-CAT films by varying the percentage of caesium, where Cs^+^ was added to FA_0.83_MA_0.17_Pb(I_0.83_Br_0.17_)_3_ to form Cs_*x*_(FA_0.83_MA0.17)_1–*x*_Pb(I_0.83_Br_0.17_)_3_, with *x* = 0%, 5%, 10%, 15% and 20%. Subsequently, the crystal phases of the films as exhibited by static GIWAXS analysis were determined. Fig. 1 ▸(*a*) shows the GIWAXS maps of the peaks when no caesium is added to the triple cation at *q* = 0.9 Å^−1^ and at *q* = 0.83 Å^−1^, corresponding to PbI_2_ and the δ-intermediate yellow phase, respectively, while the α-black phase (100) at *q* = 1.0 Å^−1^ is extremely insignificant.

However, the addition of caesium to the cation improves the crystallization process with the formation of the α-black phase at (100) conspicuously appearing at *q* = 1.0 Å^−1^ as the percentage of caesium is increased to 5%, 10%, 15% and 20% as shown in Figs. 1 ▸(*b*)–1(*e*). It is noted that 5% caesium produced the best crystal structure formation with no δ-intermediate hexagonal or PbI_2_ peaks, as compared with 10%, 15% and 20% in which the PbI_2_ peaks were produced due to excess PbI_2_ and the intermediate phase resulting from incomplete conversion to the α-cubic phase of perovskite due to large cation compositions or dense crystal points delaying the transition process. It was postulated that the films need to be annealed within the annealing window under optimized temperature conditions to avoid the intermediate phases in the process of crystallization (Qin *et al.*, 2019 ▸).

Remarkably, the addition of 5% caesium to the mixed perovskites improves the crystallization of the perovskite film (Saliba *et al.*, 2018 ▸), bypassing the hexagonal phase during the spin-coating process. It was observed during spin-coating that, when ethyl-acetate/chloro­benzene was added some 5 s before the end of the spinning process, a brown phase appeared which was responsible for the peaks at *q* = 8.6 Å^−1^, observations which were not made when Cs^+^ was not added. With a short annealing time of approximately 3–5 min the α-black phase forms. This suggests that Cs^+^ highly improves crystal formation of the desired α-black perovskite phase as reported in other studies (Mittal *et al.*, 2023 ▸; Jeon *et al.*, 2015 ▸).

### 3.2. Phase segregation on the RH probing

#### 3.2.1. Effects of RH on the 5% Cs triple cation

**Figure 2:**
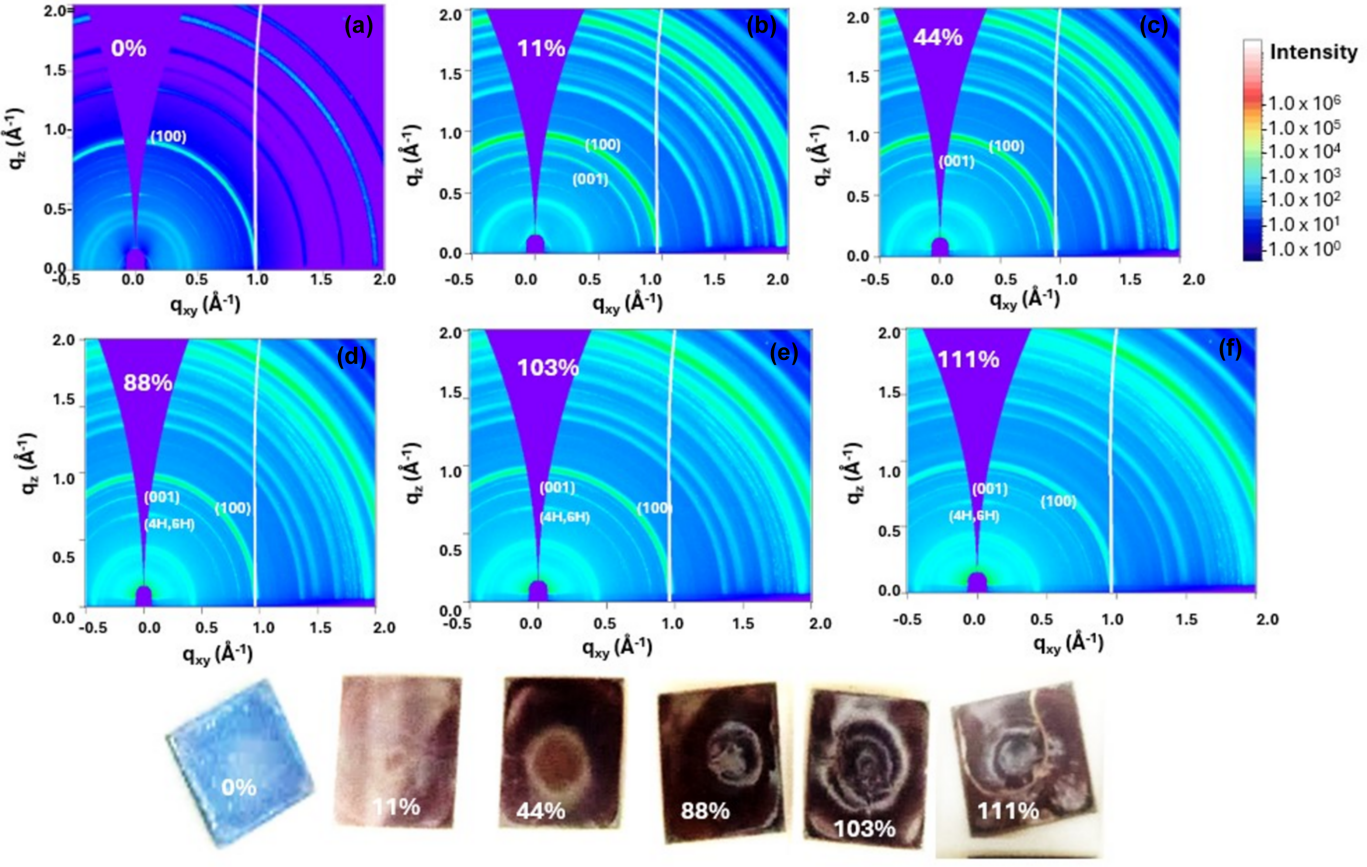
GIWAXS maps of 3-CAT upon exposure to moisture with RH values of (*a*) 0% (*b*) 11% (*c*) 44% (*d*) 88% (*e*) 103% and (*f*) 111%. Real perovskite films with respective humidities are also shown.

Fig. 2 ▸ shows GIWAXS maps for 5% Cs which is an exposition of its phase segregation when subjected to humidity. In Fig. 2 ▸(*a*) the bright peak at *q* = 1.0 Å^−1^ clearly depicts the cubic perovskite phase demonstrating no segregation to intermediate phases with no exposure to humidity. In Fig. 2 ▸(*b*) the films were exposed to low relative humidities of 11%, and parasitic peaks are immediately seen. The upshot shows a weak peak at *q* = 0.8 Å^−1^ after an exposure time of 10 s, although the intensity of the peak at *q* = 1.0 Å^−1^ was still brighter and stronger. The peak at *q* = 0.83 Å^−1^ showed that the film easily segregates slightly into the hexagonal phase, with the cubic phase remaining stable (Qin *et al.*, 2019 ▸; Dang *et al.*, 2019 ▸; Guo *et al.*, 2021 ▸).

The same results were observed with other values of RH, as shown in Fig. 2 ▸(*c*) for an RH value of 44% and in Fig. 2(*d*) for an RH value of 88%. For an RH value of 88% the peak at *q* = 0.83 Å^−1^ brightens giving more information about the phase changes. However, when the RH values were elevated beyond 100% the intensity of the peak at *q* = 1.0 Å^−1^ lost its strength and became weaker. There was accretion of more peaks with a double peak appearing at *q* = 0.83 Å^−1^ and *q* = 0.9 Å^−1^. These peaks occurred because the perovskite films could no longer withstand the elevated humidity conditions hence the cubic phase disintegrates into the hexagonal phase and also sets up the PbI_2_ phase.

**Figure 3:**
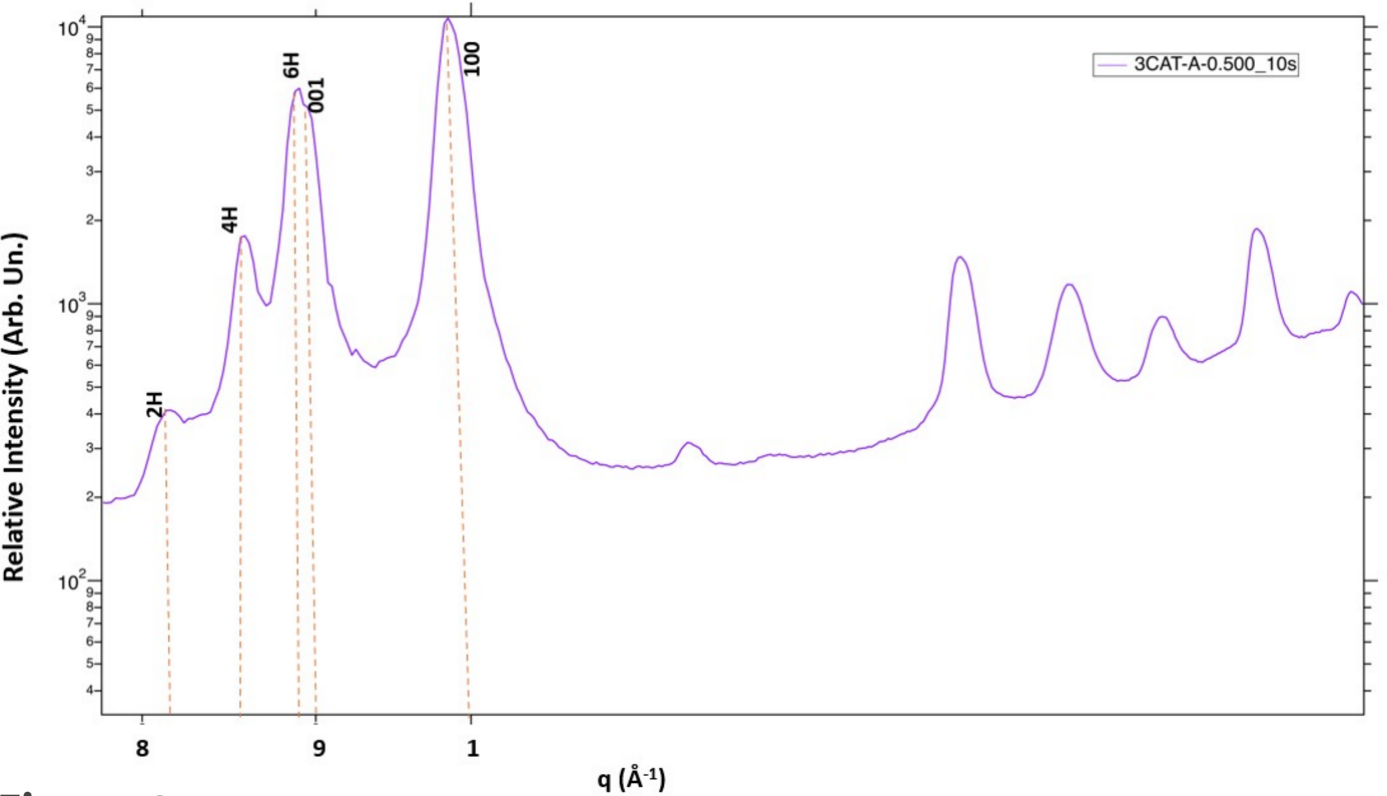
GIWAXS of 3-CAT with 5% Cs in *q*-space for RH of 111%.

Fig. 3 ▸ reports the 1D peaks drawn from the RH value of 111%, which shows a cubic perovskite (110) phase still withstanding the humid conditions at *q* = 1.0 A^−1^; however, there are accompanying crystal planes at *q* = 0.83 Å^−1^ (2H), double peaks at *q* = 0.86 Å^−1^ and 0.87 Å^−1^ (4H, 6H) and a minor peak at 0.89 Å^−1^ (PbI_2_), which are due to phase segregation recounting the hexagonal phases which are similar to other results reported elsewhere (Guo *et al.*, 2021 ▸; Li, Bretschneider *et al.*, 2016 ▸; Kazemi *et al.*, 2023 ▸). The segregation of 3-CAT back to intermediate phases due to humidity is attributed to hydro­philic bonds introduced during the formation of 3-CAT, making humidity quench the perovskite, leaving the film disoriented. In addition, the annealing window, anti-solvent dripping time and anti-solvent quantities are crucial for complete perovskite phase conversion and phase stability.

#### 3.2.2. Impact of RH on MAPI

**Figure 4:**
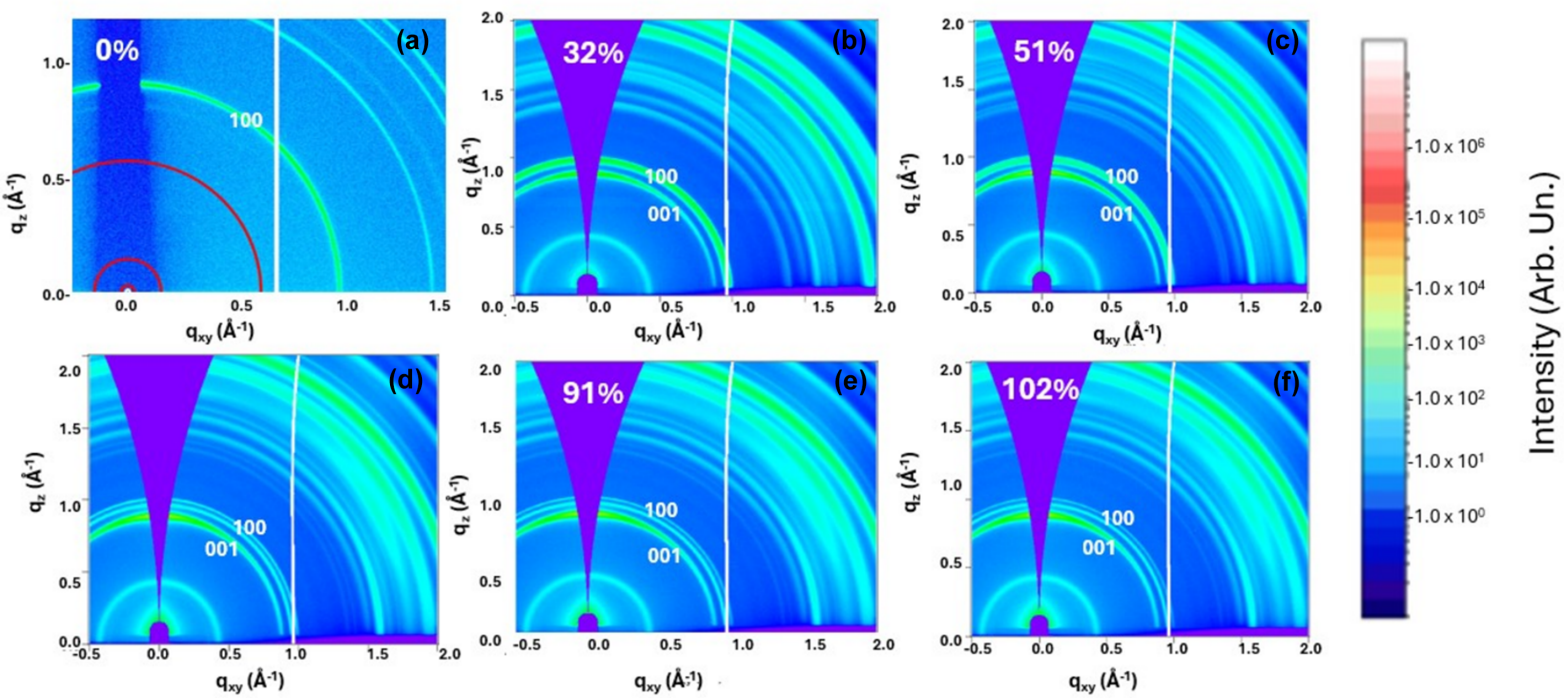
GIWAXS maps of MAPI upon exposure to moisture with RH values of (*a*) 0%, (*b*) 32%, (*c*) 51%, (*d*) 82%, (*e*) 91% and (*f*) 102%.

**Figure 5:**
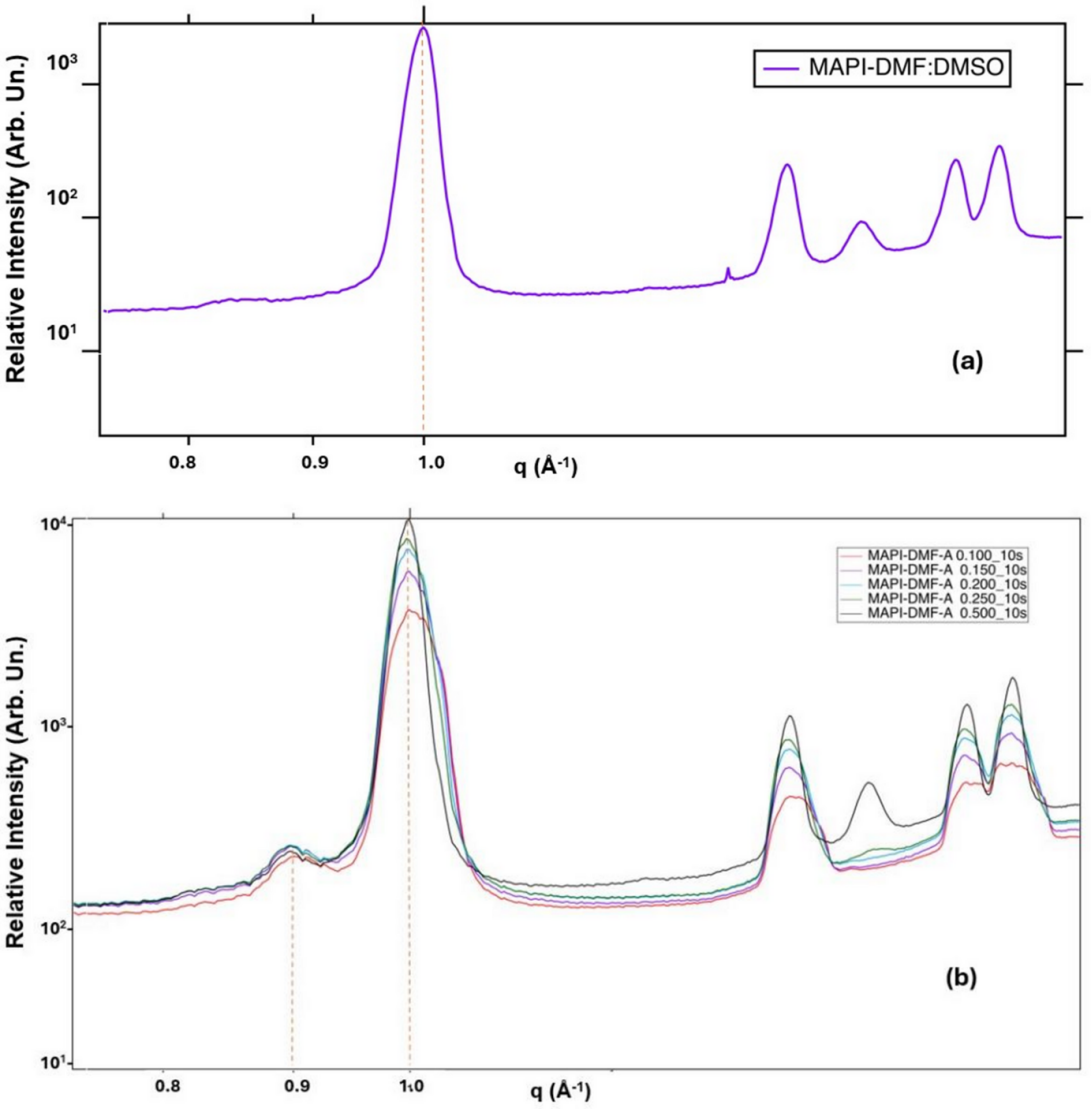
GIWAXS peaks of MAPI when exposed to 10% RH for 10 s with a slight variation of scanning points ranging from 0.100 mm to 0.500 mm starting from the edge of the sample.

We performed the same tests on MAPI, and from the maps in Fig. 4 ▸ it was evident that humidity had little influence on MAPI compared with 3-CAT, unlike temperature which vaporizes MA in MAPI whereas 3-CAT remains stable (Palazon *et al.*, 2018 ▸). The maps in Fig. 4 ▸ obtained from the GIWAXS measurements show that, without RH, MAPI does not show PbI_2_ and intermediate phases as seen in Fig. 4 ▸(*a*), which is supported by the *q*-space plot in Fig. 5 ▸(*a*). However, RH values of 32% (*b*), 51% (*c*), 82% (*d*), 92% (*e*) and 102% (*f*) for an exposure time of 10 s present distinct peaks at a *q*(*x*, *y*) value of 1.0 Å^−1^ which is a typical perovskite phase of (100) with slit peaks at *q* = 0.9 Å^−1^ corresponding to the (001) phase as seen in Fig. 4 ▸(*b*). It is also noted that, with increasing RH levels from 32% to 102%, the MAPI perovskite phase (100) remains stable with a slight shift back to the (001) phase, which is PbI_2_ at 0.9 Å^−1^, and Fig. 5 ▸(*b*) shows the PbI_2_ peaks from the minimal phase shift of MAPI. In addition, the peaks of the (100) planes appear double due to reflections caused by the crystallographic orientation normal to the substrate, resulting from varying perspectives of the spot.

However, the peaks at 0.9 Å^−1^ become clearer as RH is increased from 0% to 102%. Fig. 4 ▸(*a*) demonstrates the (100) peak of MAPI with relatively low humidity tests; the result rightly rules out predator phases in the film, building confidence for non-segregation of the film back to the (001) phases (Doherty *et al.*, 2021 ▸).

In Figs. 4 ▸(*b*)–4(*d*) the (100) perovskite peak at *q* = 1.0 Å^−1^ is still intensely seen, with other smaller peaks appearing at a *q* value of 0.9 Å^−1^. The small peak at *q* = 0.9 Å^−1^ was a result of segregation of the films slightly back to the plane (001) of the PbI_2_ phase which intensified as RH was increased to 51%. We thus deduce that, although humidity influences the crystallization of MAPI, the crystals remain slightly resistant to low values of RH stress and degrade to the PbI_2_ phase as RH values are increased to 51%, with only the (001) crystal planes appearing, comparable with other findings (Doherty *et al.*, 2021 ▸).

### 3.3. Phase identification during crystallization of MAPI and 3-CAT using micro-XRD

The static X-ray micro-diffraction technique was used to identify the crystalline structure of the films when evaporated with ethyl acetate (EA) and chloro-benzene (CB) anti-solvents at low RH conditions of 10%. The micro-XRD technique was preferred to the normal technique since it uses a narrow X-ray beam to examine a very small area of the sample, while XRD measures the entire area illuminated by the X-ray beam. This is vital in unveiling the micro-crystals in the films.

**Figure 6:**
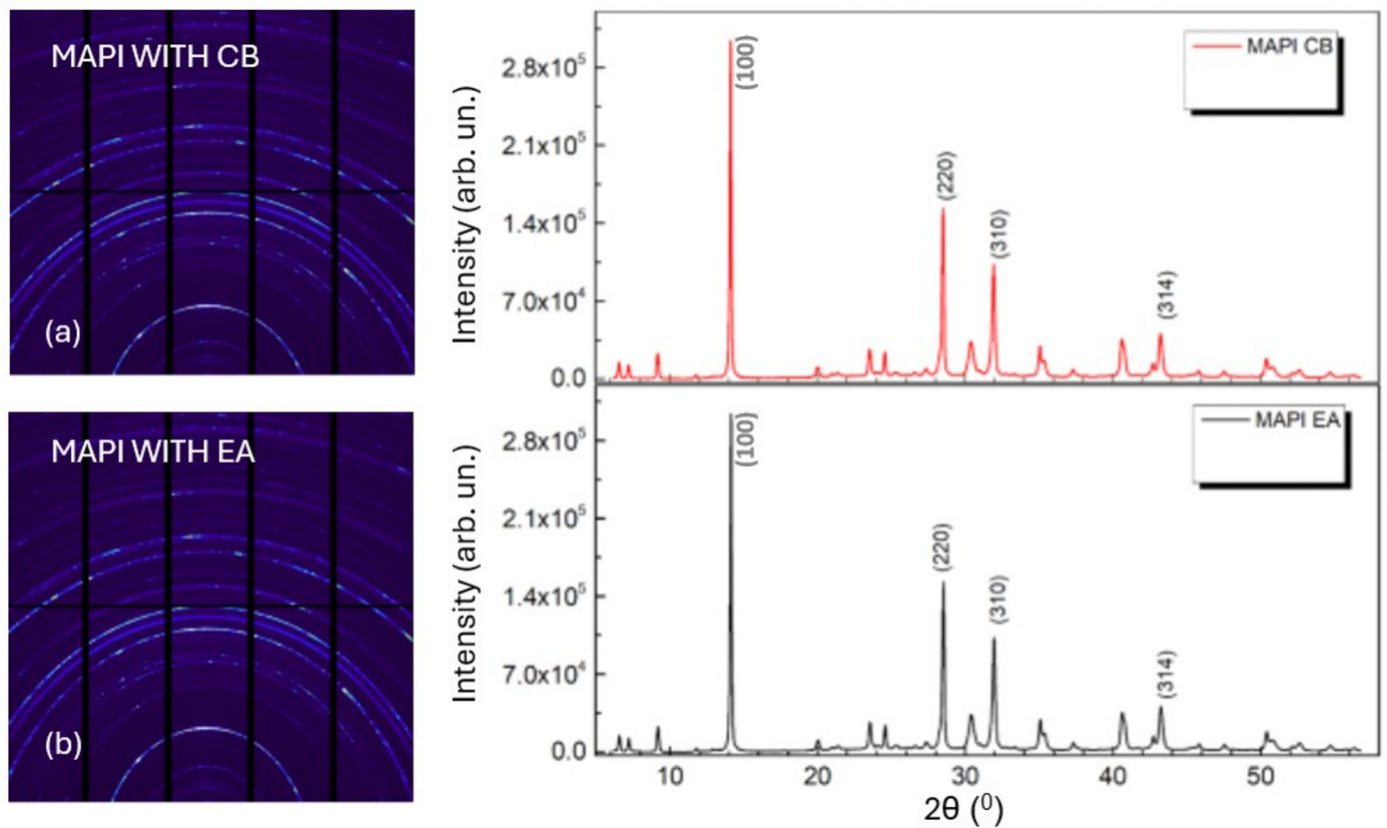
Micro-diffraction synchrotron maps for films treated with different anti-solvents. (*a*) MAPI treated with CB and the corresponding adjacent 1D peaks. (*b*) MAPI treated with EA with the adjacent 1D peaks.

It should be noted that 1D graphs have been represented in 2θ (°) space unlike the *Q*-space used in GIWAXS; this is due to the difference in the analysis tools at the two beamlines. However, the two sets of data correlate such that the crystal planes (100), (220), (310) and (314) correspond to plane angles of 14.1°, 28.5°, 34.1° and 43.6°, respectively. The pattern maps obtained for MAPI treated with both EA and CB show several peaks with typical perovskite peaks at 2θ = 14° as seen in Figs. 6 ▸(*a*) and 6(*b*). The experiment, having been performed using the anti-solvents technique, revealed that the film formation mechanism depends strongly on the anti-solvents used, with peaks delineating different planes for various anti-solvents, comparable with the peaks reported by others (Luo & Daoud, 2016 ▸).

**Figure 7:**
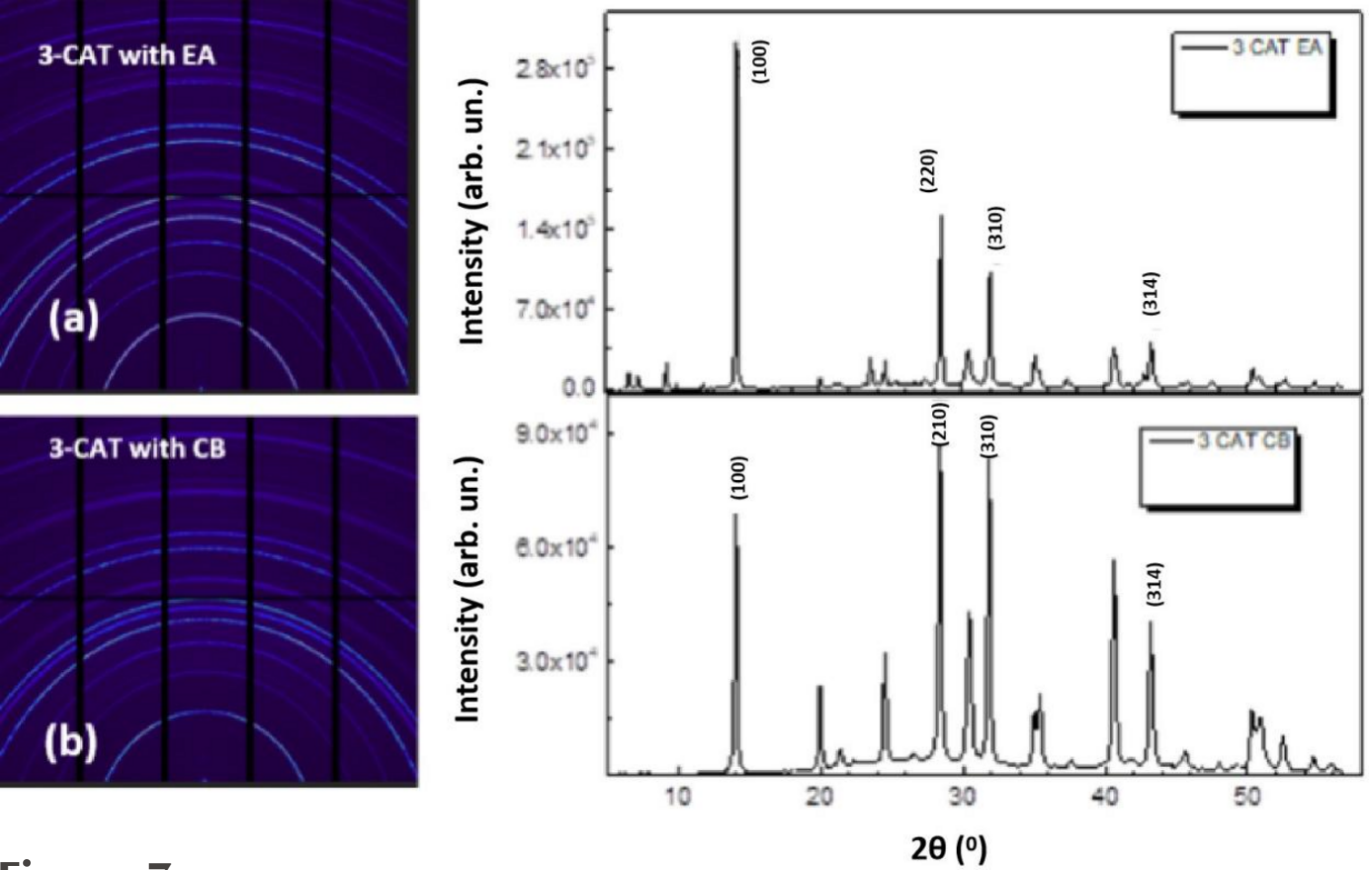
Micro-diffraction peaks for 3-CAT dissolved in both EA and CB, showing sharp peaks of the perovskite phase (100) with small peaks of crystal planes of (220), (310) and (314) corresponding to plane angles of 14.1°, 28.5°, 34.1° and 43.6°, respectively.

Pure EA used for both MAPI, as given in Fig. 6 ▸(*b*), and 3-CAT, shown in Fig. 7 ▸(*a*), produces similar peaks as with CB, shown in Figs. 6 ▸(*a*) and 7 ▸(*b*). The result illustrates typical (100) perovskite peaks with small peaks of crystal planes of (220) and (110), corresponding to plane angles of 14.1°, 28.5° and 34.1°, respectively, as discussed in other work (Zhao *et al.*, 2019 ▸). EA in Figs. 6 ▸(*b*) and 7 ▸(*a*) produces some diminished peaks at 8.3° and 9°, with similar observations being made for CB when used with MAPI as seen in Fig. 6 ▸(*a*), but the peaks tend to vanish when CB is used with 3-CAT as seen in Fig. 7 ▸(*b*). We presume this to be due to non-converted perovskite precursors (Abdur, 2015 ▸). These are parasitic peaks which introduce pinholes in the film reducing charge transport by increasing resistance. We strongly suggest that CB treatment eliminated these peaks at 8.3° and 9° for 3-CAT films and introduces more perovskite crystal planes of (112), (211), (220), (310) and (314) at 2θ° = 20°, 25°, 28.8°, 32.1° and 43°, respectively, shown in Fig. 7 ▸(*b*) and as observed elsewhere (Zhao *et al.*, 2019 ▸; Abdur, 2015 ▸; Basumatary & Agarwal, 2020 ▸; Mirhendi *et al.*, 2017 ▸).

A comparative study of the crystal planes produced for MAPI and 3-CAT as presented in Figs. 6 ▸ and 7 ▸ gives CB an edge over EA; however, there is no effect on MAPI with treatment of both EA and CB. Furthermore, the intensities of the peaks produced by EA in the 3-CAT films are lower, with maximum intensity values of 3.0 × 10^5^ a.u. (arbitrary units) seen in Figs. 7 ▸(*a*) and 7(*b*). CB, on the other hand, engendered a maximum peak of 7.0 × 10^5^ a.u. exhibited by Fig. 7 ▸(*b*). This indicates that CB leads to a greater amount of crystals with distinct spacing (https://batch.libretexts.org/print/Letter/Finished/chem-142900/Full.pdf). The peaks are also thinner, corresponding to the formation of larger crystals and larger grain size. This contributes to a longer carrier lifetime and smaller carrier transport resistance (Rong *et al.*, 2021 ▸), with credible information on the reduced recombination in the perovskite films fabricated (https://batch.libretexts.org/print/Letter/Finished/chem-142900/Full.pdf), which are beneficial for solar cell devices.

We propose optimization of the anti-solvent dropping time and quantities for EA to limit the emergence of the non-pre-perovskite phase. These studies on anti-solvent dropping conditions on 3-CAT perovskite films under inert conditions at room temperature from the results in Fig. 7 ▸ revealed that CB with the right combination of relative humidity and anti-solvent dropping time affects the (100) orientation of crystal growth resulting in pinhole-free perovskite films (Hosseinmardi *et al.*, 2020 ▸).

### 3.4. Atomic force microscopy surface analysis of 3-CAT

**Figure 8:**
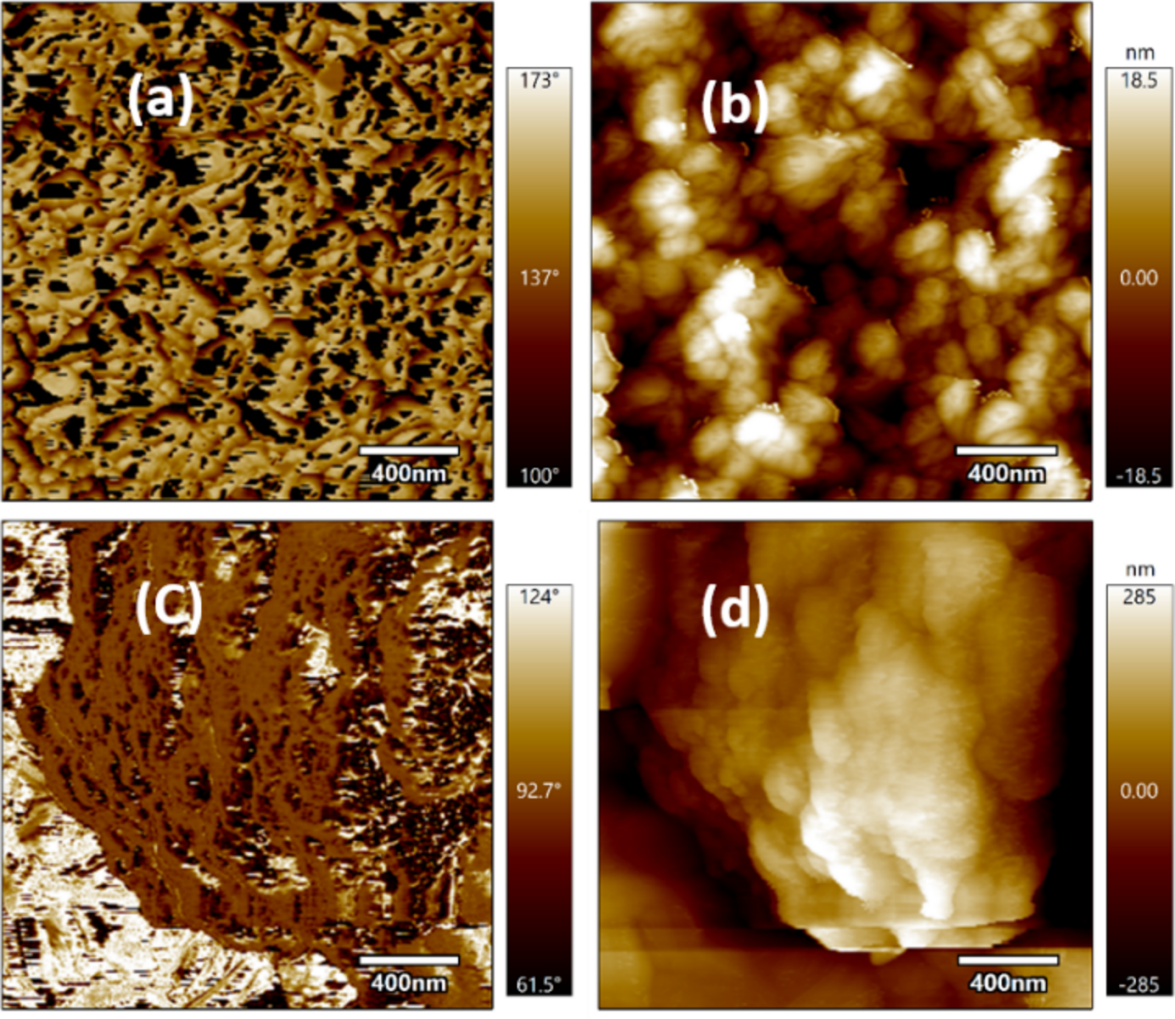
AFM topographical maps for typical perovskite surfaces: (*a*) Cs_0.05_(MA_0.17_FA_0.83_)_0.95_Pb(I_0.83_Br_0.17_)_3_ with CB, (*b*) Cs_0.05_(MA_0.17_FA_0.83_)_0.95_Pb(I_0.83_Br_0.17_)_3_ with EA, (*c*) Cs_0.10_(MA_0.17_FA_0.83_)_0.90_Pb(I_0.83_Br_0.17_)_3_ with CB, (*d*) Cs_0.10_(MA_0.17_FA_0.83_)_0.90_Pb(I_0.83_Br_0.17_)_3_ with EA.

Atomic force microscopy (AFM) topographical maps for Cs_*x*_(MA_0.17_FA_0.83_)_1–*x*_Pb(I_0.83_Br_0.17_)_3_, *x* = 0.05 and 0.10, are presented in Fig. 8 ▸. Triple cation perovskites demonstrated good topographical surface morphologies which is paramount for high efficiencies. The data revealed the root-mean-square (RMS) roughness of average grain size 18.5 nm and 28.5 nm for *x* = 0.05 and 0.10, respectively. The results reveal smooth films similar to the data presented by Cui *et al.* (2016 ▸); their topography data showed root-mean-square (RMS) roughnesses of 20.3 nm, 27.8 nm, 19.2 nm and 22.7 nm for colored films of MAPbI_3_, MAPbBrI_2_, MAPbBr_2_I and MAPbBr_3_, respectively.

Similar findings were presented for the morphology of 3D/2D perovskite films studied by Abbas *et al.* (2020 ▸) using AFM in tapping mode, showing that 3D/2D films have smoother surfaces and longer grain size compared with pristine perovskite film. They opined that AFM images for FA^+^ and Cs^+^ (a total of three cations: MA^+^, An^+^ and FA^+^, or Cs^+^, ions in the molar ratio 0.8:0.15:0.5, respectively, in the perovskite precursor solution) displayed smoother perovskite films with crystal grain size larger than pure 3D MAPbI_3_ but fractionally less than 2D/3D perovskites without FA^+^ or Cs^+^ ions. The AFM data we obtained also agree with this work, in which the addition of Cs ions in the MA^+^ >and FA^+^ cations increases the crystallization of the perovskites improving the general surface morphology of the perovskite. Cs^+^ compacts the ions reducing surface voids and further reducing the crystal grain size resulting in high functionality (Zhang *et al.*, 2018 ▸; Chen *et al.*, 2020 ▸).

We treated the films with CB and EA anti-solvents. Fig. 8 ▸(*a*) shows topographical maps for when 5% 3-CAT perovskite was treated with CB anti-solvent, while Fig. 8(*b*) shows it treated with EA. These results demonstrated smoother films obtained with EA treatment compared with CB. We treated 3-CAT perovskite films with 10% Cs added; similar results were found as depicted in Fig. 8 ▸(*c*) for CB and Fig. 8 ▸(*d*) for EA, with EA still producing smoother films. When the result is compared with the findings of Paek *et al.* (2017 ▸), who discussed the treatment of perovskite films with five different anti-solvents – tri­fluoro toluene (TFT), toluene, CB, xylene and ether, and without any treatment – it was found that TFT produces smoother films compared with CB. Furthermore, AFM images of FA_0.3_MA_0.7_PbI_2.46_Br_0.54_ and Cs_0.05_FA_0.3_MA_0.7_PbI_2.51_Br_0.54_ perovskite films on ITO/306 PEDOT:PSS substrates showed a root-mean-square (RMS) value of 11.85 nm (Pang *et al.*, 2018 ▸). Introduction of Cs ions resulted in an increase of the RMS value of Cs_0.05_FA_0.3_MA_0.7_PbI_2.51_Br_0.54_ to 12.37 nm. This explains why the use of EA smoothens the films, although CB has good crystallization peaks for perovskite application.

### 3.5. Scanning electron microscopy grain size analysis for MAPI and 3-CAT

**Figure 9:**
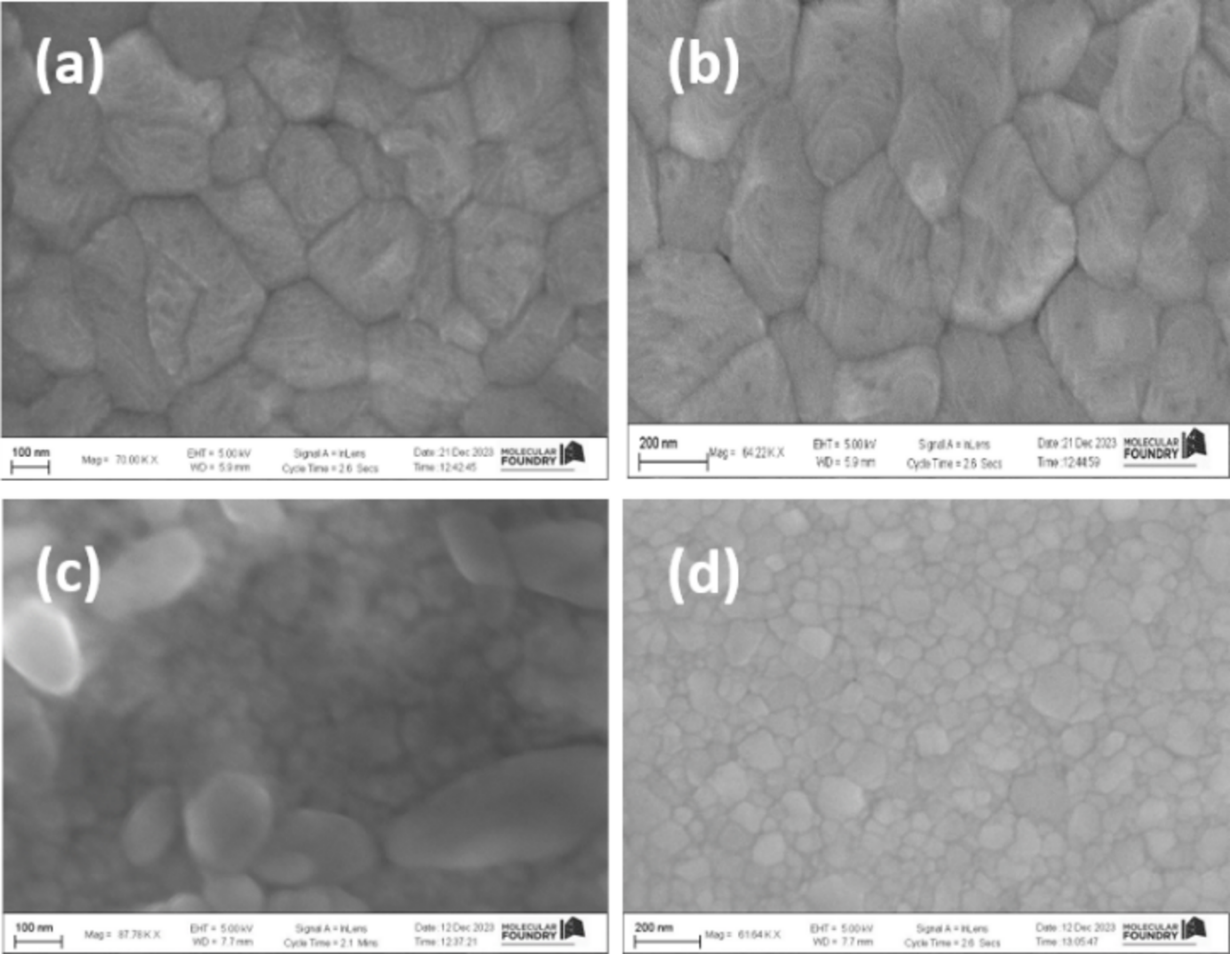
SEM maps for typical 3-CAT materials. (*a*) MAPI with CB. (*b*) MAPI with EA. (*c*) 5% Cs 3-CAT CB. (*d*) 5% Cs 3-CAT EA.

The grain size and diffusion length of the perovskite crystals were studied by scanning electron microscopy (SEM) analysis for both CB and EA treatment, demonstrating that MAPI generated larger crystal grains unlike 3-CAT, as can be seen in Figs. 9 ▸(*a*) and 9(*c*), respectively, for a magnification factor of 100. Similar results are obtained for a magnification factor of 200, shown in Figs. 9 ▸(*b*) and 9(*d*). This is attributed to a decrease in the crystal sizes after introduction of Cs ions, as observed from their SEM images (Zhang *et al.*, 2018 ▸; Chen *et al.*, 2020 ▸). The decrease in the grain sizes was due to the slow conversion of the non-perovskite hexagonal phase to the cubic perovskite phase (Ren *et al.*, 2016 ▸; Muscarella *et al.*, 2019 ▸; Chiang & Wu, 2016 ▸). However, MAPI demonstrates larger grain sizes due to faster perovskite phase conversion.

We performed SEM morphological characterization on MAPI and 3-CAT of 5% Cs to determine the shape, crystal size and coverage of the films with CB and EA solvent treatment. The grain size changes significantly from a single cation perovskite (MAPI) to triple cation (3-CAT).

Cross-sectional images showed that the triple cation film is dense and compact whereas MAPI films presented a less intense crystal structure, as can be seen in Figs. 9 ▸(*a*) and 9(*b*). On the contrary, 3-CAT has mixed crystal sizes, some being larger than others, as shown in Fig. 9 ▸(*c*). The large crystals similar to MAPI demonstrate the converted phase to cubic perovskite structure, while the smaller crystals show the non-converted phases of PbI_2_ and hexagonal perovskite phases. These results support the findings obtained from synchrotron data.

Thus 3-CAT perovskites are highly important for commercialization of perovskite solar cells. However, the fabrication parameters, including solvent dripping time and anti-solvent quantities, and an annealing window to optimize the phase conversion are paramount to realize this dream.

## 4. Conclusion

GIWAXS analysis on a triple cation perovskite showed that the addition of 5% caesium produced the best crystal structure formation with no δ-intermediate hexagonal or PbI_2_ peaks, as compared with 10%, 15% and 20% caesium addition.

Humidity has a strong influence on the crystallization of MAPI; crystals remain slightly resistant to low values of RH stress and degrade to the PbI_2_ phase as RH values are increased, with only the (001) crystal planes appearing. When 3-CAT was subjected to RH, and the values elevated beyond 100%, the intensity of the peak at *q* = 1.0 Å^−1^ became weaker. There is accretion of more peaks with a double peak appearing at *q* = 0.83 Å^−1^ and *q* = 0.9 Å^−1^. These peaks occurred because the perovskite films could no longer withstand the elevated humidity conditions, hence the cubic phase disintegrated into the hexagonal and PbI_2_ phase.

The micro-XRD pattern maps obtained for both MAPI and 3-CAT treated in EA and CB show typical perovskite peaks at 2θ = 14°. A comparative analysis of the crystal planes produced for MAPI and 3-CAT gives CB an edge over EA; however, there is no effect on MAPI with treatment of both EA and CB.

AFM results show that EA produces smooth films compared with CB, which could be the best candidate for perovskites except for its extra peaks produced during film formation. MAPI produced larger films than 3-CAT from the SEM findings. This is attributed to slow conversion of the hexagonal phase to the cubic perovskite phase although with a compact structure. This was postulated due to the increased crystal size.

## 5. Outlook

Complex information, real-time structural evolution and knowledge of the crystallization pathways of 3-CAT by both *in situ* and *ex situ* GIWAXS measurements still need further research. For instance, understanding the crystallization kinetics of 3-CAT is rather difficult due to the stoichiometry of the perovskite layer that can be better preserved by applying anti-solvent via carrier-gas-free spraying (Telschow *et al.*, 2022 ▸). By exploring different volumes of anti-solvent spraying, as little as 60 µL may result in more stable perovskite films.

A good understanding of the stoichiometry, dissolving mechanics, solvent administering and annealing procedure is cardinal in achieving the desired results in the more efficient 3-CAT perovskites. We thus propose optimization of film formation parameters, including annealing time, anti-solvent dripping time and anti-solvent quantities, in future work which contribute to complete triple cation perovskite conversion.
